# Identification of a diarylpentanoid-producing polyketide synthase in the biosynthesis of 2-(2-phenylethyl)chromones in agarwood

**DOI:** 10.1007/s11418-023-01743-5

**Published:** 2023-08-19

**Authors:** Hiroyuki Morita, Yuan-E Lee, She-Po Shi

**Affiliations:** 1 Institute of Natural Medicine, University of Toyama, 2630-Sugitani, Toyama, 930-0194 Japan; 2 Modern Research Center for Traditional Chinese Medicine, Beijing University of Chinese Medicine, Beijing, 100029 People’s Republic of China

**Keywords:** Agarwood, Type III polyketide synthase, *Aquilaria sinensis*, Biosynthesis

## Abstract

Agarwood has been valued as an exquisite, high-grade fragrant wood since ancient times. Due to the scarcity of high-quality agarwood, it is quite expensive, and the number of original plants has been drastically reduced due to overharvesting, including illegal logging. Despite this, a reliable method of agarwood cultivation has yet to be developed. Thus, identifying the biosynthetic pathways of the fragrant components in agarwood might help developers to optimize the culture conditions and create artificial agarwood, by monitoring the expression of the biosynthetic enzymes or their genes. This review presents the characteristics of our recently identified key enzyme, 2-(2-phenylethyl)chromone precursor synthase (PECPS), which generates the common precursor of 2-(2-phenylethyl)chromones (PECs), the main fragrances in agarwood, as well as our reasoning to reach these conclusions. We also discuss the biosynthetic pathway of PECs, unveiled following the identification of PECPS.

**Supplementary Information:**

The online version contains supplementary material available at 10.1007/s11418-023-01743-5.

## Introduction

**Fig. 1:**
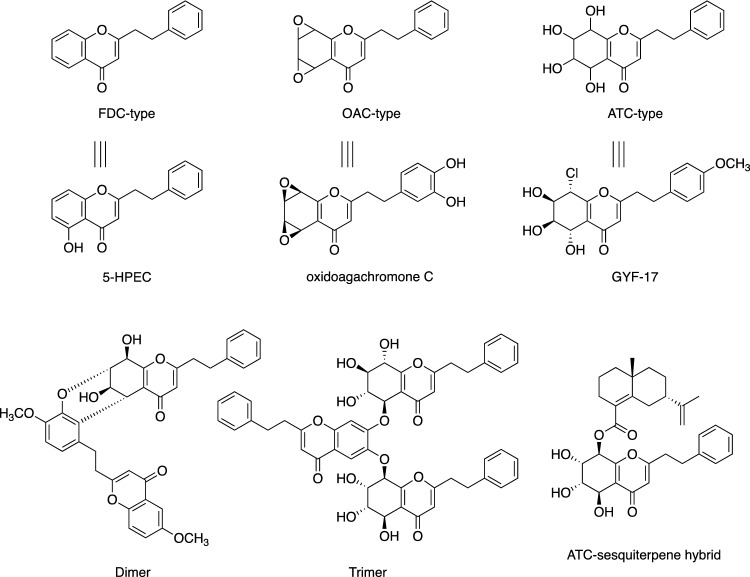
Examples of the structures of PECs isolated from agarwood

Agarwood, also known as aloeswood, gaharu, eaglewood, jinkoh and chenxiang, is a highly prized, expensive wood. For centuries, it has been used in China, Japan, and other countries as an exquisitely fragrant wood [1–4]. Agarwood is also quite popular in Japan, where it is used as incense in traditional ceremonies. Agarwood is valued not only as a fragrance but also as a digestive, analgesic, and antiemetic agent to treat abdominal pain, vomiting, and insomnia, in a wide range of Asian countries [2]. The original agarwood plants are *Aquilaria sinensis*, *A. malaccensis*, and *A. agallocha*, which belong to the family Thymelaeaceae, and the heartwood of their wood or root is used as agarwood. The principal components of agarwood are 2-(2-phenylethyl)chromones (PECs) [5–22]. The PEC monomers are structurally classified into three types, flindersiachromones (FDC), oxidoagarochromones (OAC), and agarotetrolchromones (ATC), and these monomers, as well as their dimers and trimers, and the hybrid types combined with sesquiterpenes, have been identified as the aromatic components of agarwood (Fig. 1). The exquisite fragrance of agarwood is thought to be produced by the synergistic effects of these slightly different fragrances and sesquiterpenes, which are the essential oil components. Recently, PECs have also attracted attention from a pharmaceutical perspective due to their physiological activities, including neuroprotective, acetylcholinesterase inhibitory, anti-inflammatory, and antibacterial effects [23–31]. However, these PECs are grouped in compounds that accumulate within resin at parts of the wood damaged by prolonged pathological stress, such as bacterial infection and insect infestation. In other words, the original plants on the verge of death are considered to be agarwood, and thus only a few percent can be used. As a side note, the name “agarwood” is derived from the fact that the wood becomes submerged in water due to its increased specific gravity by the deposition of resin.

To produce agarwood artificially, various efforts such as artificially stressing the original plant have been attempted in some countries, including Thailand, Vietnam, and Myanmar, but in spite of the cost and labor involved, such products are generally of inferior quality and not always successfully recognized as agarwood. Although the authors have not directly seen the method, artificial agarwood is cultivated by hammering nails into the trunks of  ~ 10-year old original trees, drilling holes in them, and inoculating specific bacteria into the drilled holes, which then stimulate resin production [32–36]. Furthermore, attempts to produce the most expensive type of agarwood, which can be traded at prices higher than the same weight of gold and requires the accumulation of resin over many years, have been unsuccessful so far. Due to the rarity of agarwood, the market price is always extravagant. Therefore, illegal logging has persisted and the number of original plants has been dramatically reduced. Accordingly, the authors have been conducting research on the biosynthesis of agarwood PECs, to develop a new methodology for the cultivation of high quality, artificial agarwood.

In this paper, we review the recent advances in biosynthetic studies of PECs in agarwood, obtained during the course of our research. We demonstrate that PECs are biosynthesized from a common precursor with a C_6_–C_5_–C_6_ skeleton produced by 2-(2-phenylethyl)chromone precursor synthase (PECPS), a type III polyketide synthase (PKS), which is mechanistically distinct from other previously reported type III PKSs.

### Identification of the gene encoding the enzyme involved in the 2-(2-phenylethyl)chromone scaffold biosynthesis

**Fig. 2:**
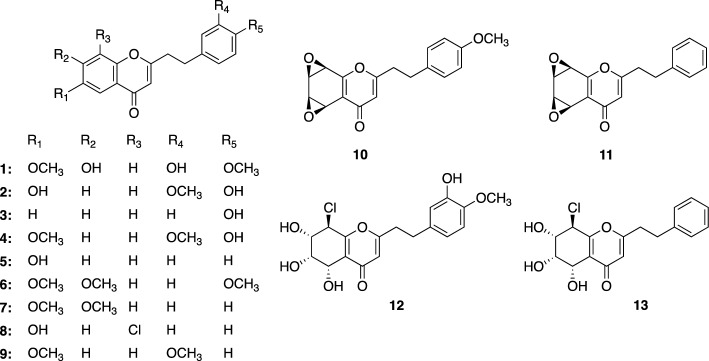
Structures of PECs isolated from *A. sinensis* calli

**Fig. 3:**
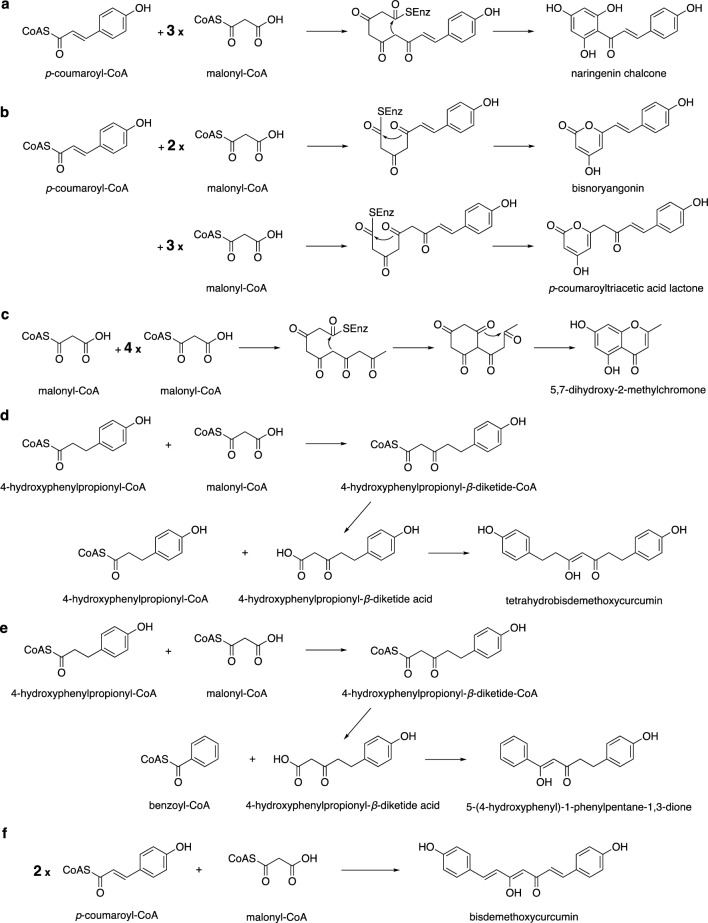
Formation of polyketides by type III PKSs. **a‒f** Proposed synthetic mechanisms of **a** naringenin chalcone by CHS including AsCHS, **b** bisnoryangonin and *p*-coumaroyltriacetic acid lactone by AsPKS1 and AsPKS2, **c** 5,7-dihydroxy-2-methylchromone by PCS, **d** tetrahydrobisdemethoxycurcumin by the CUS Met265Val and Gly274Phe mutants and PECPS, **e** 5-(4-hydroxyphenyl)-1-phenylpentane-1,3-dione by PECPS, and **f** bisdemethoxycurcumin by CUS

Most candidate genes responsible for the biosynthesis of natural compounds in plants have been identified by investigating the enzyme genes expressed in response to the accumulation of the target natural organic compounds, and then analyzing the functions of the candidate genes. This general methodology was also used in this study. However, to experimentally predict the biosynthetic genes using this method, the efficient analysis of the gene expression linked to the accumulation of compounds is required. Therefore, using plants as the target of this study is not feasible, due to their slow growth rate. To this end, Professor Shi (one of the authors of this review) and his group first created *A. sinensis* calli that successfully produced PECs (**1**‒**13**), especially the FDC-type, under salt stress conditions (Fig. 2) [37]. The key enzymes involved in PECs biosynthesis were predicted to be type III PKSs, since the structures of PECs are similar to those of flavonoids such as apigenin. In addition, chalcone, the common flavonoid biosynthetic precursor with a C_6_–C_3_–C_6_ skeleton, is biosynthesized by chalcone synthase (CHS), a type III PKS family member (Fig. 3a) [38–40].

**Fig. 4:**
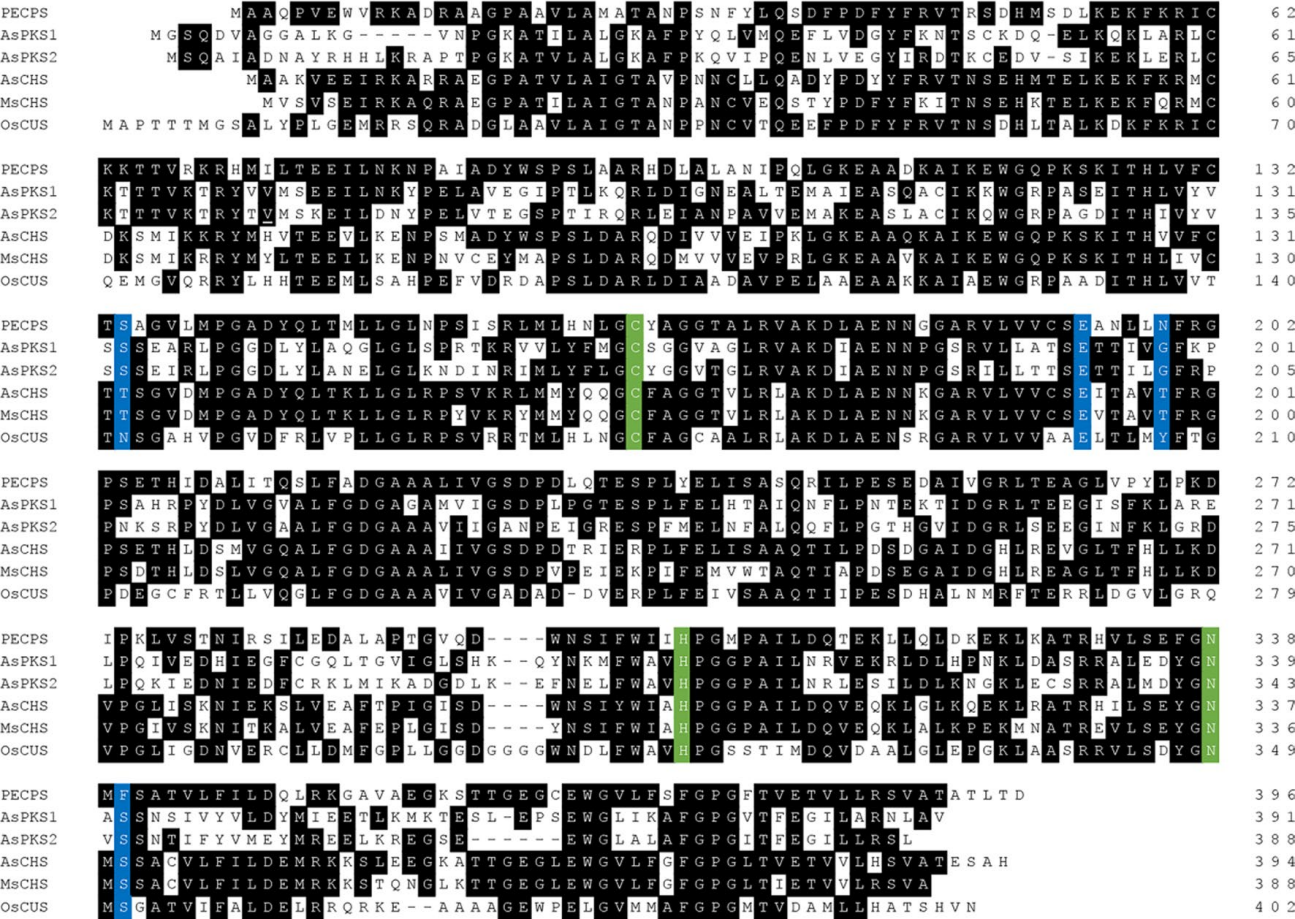
Comparisons of amino acid sequences of *A. sinensis* PKSs and other type III PKSs. The Cys, His, and Asn catalytic triad residues are colored green. The residues forming the Ser351-Asn142-H_2_O-Tyr207-Glu202 rearrangement in CUS and the corresponding residues in other type III PKSs are highlighted in blue. GenBank accession numbers are as follows: PECPS, MH885494; AsPKS1, MW380862; AsPKS2, MW380863; AsCHS, EF103197; MsCHS (*Medicago sativa* CHS2), L02902; OsCUS, BAC79571. PECPS shares 39%, 37%, 65%, 60%, and 47% amino acid identities with AsPKS1, AsPKS2, AsCHS, MsCHS, and OsCUS, respectively

Investigations of the type III PKS genes in the calli suggested that the expression of genes encoding four type III PKSs (PECPS, AsPKS1, AsPKS2, and AsCHS) was upregulated in response to the accumulation of the aforementioned PECs [41, 42]. Their amino acid sequences revealed the conservation of the catalytic triad, cysteine (Cys), histidine (His), and asparagine (Asn), which is crucial for the condensation reaction by type III PKSs (Fig. 4) [38–40, 43]. A phylogenetic tree analysis demonstrated that AsCHS belongs to the same subgroup as CHS, whereas PECPS, AsPKS1, and AsPKS2 belong to different subgroups, and thus one of the PECPS, AsPKS1, and AsPKS2 enzymes, or a combination, may be involved in the formation of the PECs scaffold [41, 42].

To further clarify each enzyme function, AsCHS, belonging to the CHS family in the phylogenetic tree, and AsPKS1 and AsPKS2, which are located furthest away from CHS in the phylogenetic tree, were expressed in *Escherichia coli*, and their enzymatic reaction products derived from *p*-coumaroyl-CoA and malonyl-CoA were investigated [41, 42]. As expected, AsCHS produced naringenin chalcone from the condensations of three malonyl-CoAs with *p*-coumaroyl-CoA via Claisen-type cyclization, confirming its function as a CHS (Fig. 3a). In contrast, both AsPKS1 and AsPKS2 produced α-pyrones, bisnoryangonin and *p*-coumaroyltriacetic acid lactone, derived from two and three malonyl-CoAs with *p*-coumaroyl-CoA via lactonization, respectively (Fig. 3b), which are known as byproducts of the in vitro CHS reactions [40]. The structures of α-pyrones are unlikely to be components of the PEC scaffold. Therefore, these results strongly suggested that PECPS might be the target type III PKS involved in the biosynthesis of the PEC scaffold. However, it was difficult to predict its functions solely from the amino acid sequence.

### Functional analysis of PECPS

Considering the structures of PECs, their scaffolds may be biosynthesized from the condensations of four malonyl-CoAs with 4-hydroxyphenylpropionyl-CoA via the formation of a chromone ring, in a manner similar to that of pentaketide chromone synthase (PCS) from *Aloe arborescens* (Fig. 3c and Supplementary Fig. S1) [44, 45]. Thus, PECPS was heterologously expressed in *E. coli* and the products derived from two CoA-thioester substrates, 4-hydroxyphenylpropionyl-CoA and malonyl-CoA, were investigated in the presence of purified recombinant PECPS. Unexpectedly, PECPS produced tetrahydrobisdemethoxycurcumin, which was previously reported as a diarylheptanoid generated from two 4-hydroxyphenylpropionyl-CoAs and one malonyl-CoA by the curcuminoid synthase (CUS) Met265Val and Gly274Phe mutants [46] (Fig. 3d). In light of this result, 4-hydroxyphenylpropionyl-CoA, malonyl-CoA, and benzoyl-CoA are regarded as the possible substrates of PECPS, which lead to the formation of the C_6_–C_5_–C_6_ precursor of the PEC scaffold (Fig. 3e and Supplementary Fig. S2). Furthermore, salicyloyl-CoA may also be a possible alternative substrate in place of benzoyl-CoA, and would directly lead to the formation of a chromone corresponding to the PEC scaffold (Supplementary Fig. S3). In nature, curcumin in *Curcuma longa* and alkyl quinolines in *Evodia rutaecarpa* were reportedly biosynthesized from two feruloyl-CoAs and one malonyl-CoA, and from one *N*-methylanthraniloyl-CoA, one malonyl-CoA, and one fatty acyl-CoA, respectively, by the collaboration of two functionally distinct type III PKSs [47–49]. Furthermore, although the in vivo function is still unclear, the *cus* gene has also been identified in the genome of the rice *Oryza sativa*, and an in vitro functional analysis revealed that this enzyme is capable of performing the one-pot synthesis of bisdemethoxycurcumin, by catalyzing the condensation of a malonyl-CoA with *p*-coumaroyl-CoA, followed by the condensation of the resulting *p*-coumaroyl-*β*-diketide acid with a second *p*-coumaroyl-CoA (Fig. 3f) [46, 50]. Thus, it would not be surprising that with PECPS alone, the PEC scaffold could be generated from three substrates: 4-hydroxyphenylpropionyl-CoA, malonyl-CoA, and benzoyl-CoA. Although not mentioned in our first publication on PECPS [42], we have also confirmed that AsCHS, AsPKS1, and AsPKS2 are unable to produce tetrahydrobisdemethoxycurcumin from 4-hydroxyphenylpropionyl-CoA and malonyl-CoA. Guided by this information, we subjected PECPS to an enzyme reaction using 4-hydroxyphenylpropionyl-CoA, malonyl-CoA, and benzoyl-CoA as substrates, and found that PECPS indeed produces a diarylpentanoid, 5-(4-hydroxyphenyl)-1-phenylpentane-1,3-dione, which corresponds to the precursor of the PEC scaffold (Fig. 3e and Supplementary Fig. S2) [42]. In contrast, when salicyloyl-CoA was provided instead of benzoyl-CoA, PECPS no longer produced the diarylpentanoid and the chromones corresponding to the PEC scaffold. These observations strongly suggested that PECPS is a type III PKS that biosynthesizes a linear diarylpentanoid, which would presumably be converted to the chromone form by other enzyme(s) in *A. sinensis*.

**Fig. 5:**
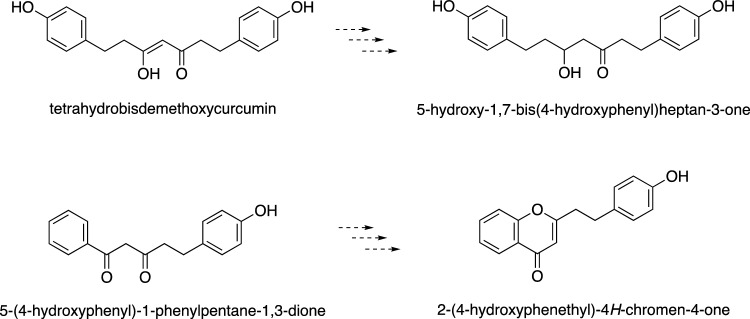
Structures of isolated compounds accumulated in *N. benthamiana* leaves with the transient expression of PECPS

To further verify the in vivo biochemical PECPS function, we conducted two in vivo experiments, the transient expression of PECPS in the model plant, *Nicotiana benthamiana*, and the knockdown expression of PECPS in *A. sinensis* calli, since the observed enzyme activity of PECPS may be limited in the in vitro enzymatic reactions [42]. The transient expression of PECPS in *N. benthamiana* revealed that 5-hydroxy-1,7-bis(4-hydroxyphenyl)heptan-3-one, a diarylheptanoid, only accumulated in leaves expressing the *pecps* gene (Fig. 5). 5-Hydroxy-1,7-bis(4-hydroxyphenyl)heptan-3-one is a tetrahydrobisdemethoxycurcumin derivative with a reduced double bond, suggesting that PECPS produces tetrahydrobisdemethoxycurcumin, and then an oxidoreductase(s) or other enzyme(s) in *N. benthamiana* presumably converts it to 5-hydroxy-1,7-bis(4-hydroxyphenyl)heptan-3-one. Furthermore, we found that the PECPS in expressed *N. benthamiana* leaves conferred the ability to produce 2-(4-hydroxyphenylethyl)-4*H*-chromen-4-one when benzoyl-CoA or 5-(4-hydroxyphenyl)-1-phenylpentane-1,3-dione was provided in the culture medium (Fig. 5). These observations demonstrated that PECPS was able to produce 5-(4-hydroxyphenyl)-1-phenylpentane-1,3-dione even in *N. benthamiana* leaves, and that the PECPS product was converted to 2-(4-hydroxyphenylethyl)-4*H*-chromen-4-one by hydroxylase(s) and/or reductase(s) that are originally present in the *N. benthamiana* leaves. In addition, an RNAi knockdown of PECPS expression in *A. sinensis* calli indicated that the production of PECs was dramatically reduced in calli that showed significantly downregulated expression of PECPS, although we were unable to completely knock out the *pecps* gene in the calli. These studies confirmed that PECPS is indeed a key biosynthetic enzyme that forms the C_6_–C_5_–C_6_ precursor of the PEC scaffold in *A. sinensis*, and that the PECs would be biosynthesized via the formation of a diarylpentanoid, 5-(4-hydroxyphenyl)-1-phenylpentane-1,3-dione.

### Catalytic mechanism of PECPS

PECPS was thus found to be a novel type III PKS that condenses 4-hydroxyphenylpropionyl-CoA, benzoyl-CoA, and malonyl-CoA to form the diarylpentanoid. From the structure of the product, PECPS is suggested to (1) accept either 4-hydroxyphenylpropionyl-CoA or benzoyl-CoA as the initial starter substrate with malonyl-CoA as the first extender substrate to produce the corresponding acyl-*β*-diketide-CoA intermediate, (2) hydrolyze the acyl-*β*-diketide-CoA to form the corresponding acyl-*β*-diketide acid intermediate as the second extender substrate, and (3) condense the acyl-*β*-diketide acid intermediate with benzoyl-CoA (when 4-hydroxyphenylpropionyl-CoA was first used) or 4-hydroxyphenylpropionyl-CoA (when benzoyl-CoA was first used), to generate a diarylpentanoid as the final product (Supplementary Fig. S2). However, the aforementioned results did not allow us to determine which substrate PECPS uses as the first starter substrate and whether PECPS is capable of catalyzing all reactions. To this end, we conducted additional experiments, including an isothermal titration calorimetry (ITC) analysis for the substrate binding affinity, LC–MS analysis for *β*-diketide-CoA and its acid formations, and a feeding experiment for the PECPS catalytic ability [42]. Notably, the ITC analysis demonstrated that the binding affinity of PECPS to 4-hydroxyphenylpropionyl-CoA (K_D_ = 18.57 ± 1.37 μM) was significantly higher than that of benzoyl-CoA (K_D_ value close to 1,000 μM). Furthermore, the LC–MS analysis revealed the appearances of 4-hydroxyphenylpropionyl-*β*-diketide-CoA and 4-hydroxyphenylpropionyl-*β*-diketide acid in the PECPS reaction products, without any evidence of the production of not only benzoyl-*β*-diketide-CoA but also benzoyl-*β*-diketide acid, suggesting that PECPS might use 4-hydroxyphenylpropionyl-CoA and malonyl-CoA to generate the corresponding *β*-diketide-CoA intermediate. The in vitro feeding experiment of 4-hydroxyphenylpropionyl-*β*-diketide-CoA in the presence and absence of PECPS indicated no differences in the amounts of 4-hydroxyphenylpropionyl-*β*-diketide acid produced by both reactions, suggesting that PECPS could not form the *β*-diketide acid intermediate from the 4-hydroxyphenylpropionyl-*β*-diketide-CoA intermediate.

It is remarkable that PECPS can catalyze the one-pot formation of the diarylpentanoid, despite its inability to cleave the thioester-bond of the *β*-diketide-CoA intermediate. We speculated that the detailed catalytic mechanism of PECPS also differs from that of CUS, which has been proposed to employ the hydrolysis reaction to generate the *β*-diketide acid, with a nucleophilic water molecule forming the hydrogen bond networks with the rearrangement of Ser351-Asn142-H_2_O-Tyr207-Glu202, neighboring the catalytic Cys at the active-site center, and to retain the *β*-diketide acid in the active-site pocket during the one-pot formation of the diarylheptanoid [46]. Thus, PECPS might also be a mechanistically new type III PKS that has never been observed previously, and this hypothesis was further verified from the X-ray crystal structure analysis of PECPS at 1.98 Å resolution, in combination with docking simulations of the substrates and putative intermediates [42].

**Fig. 6:**
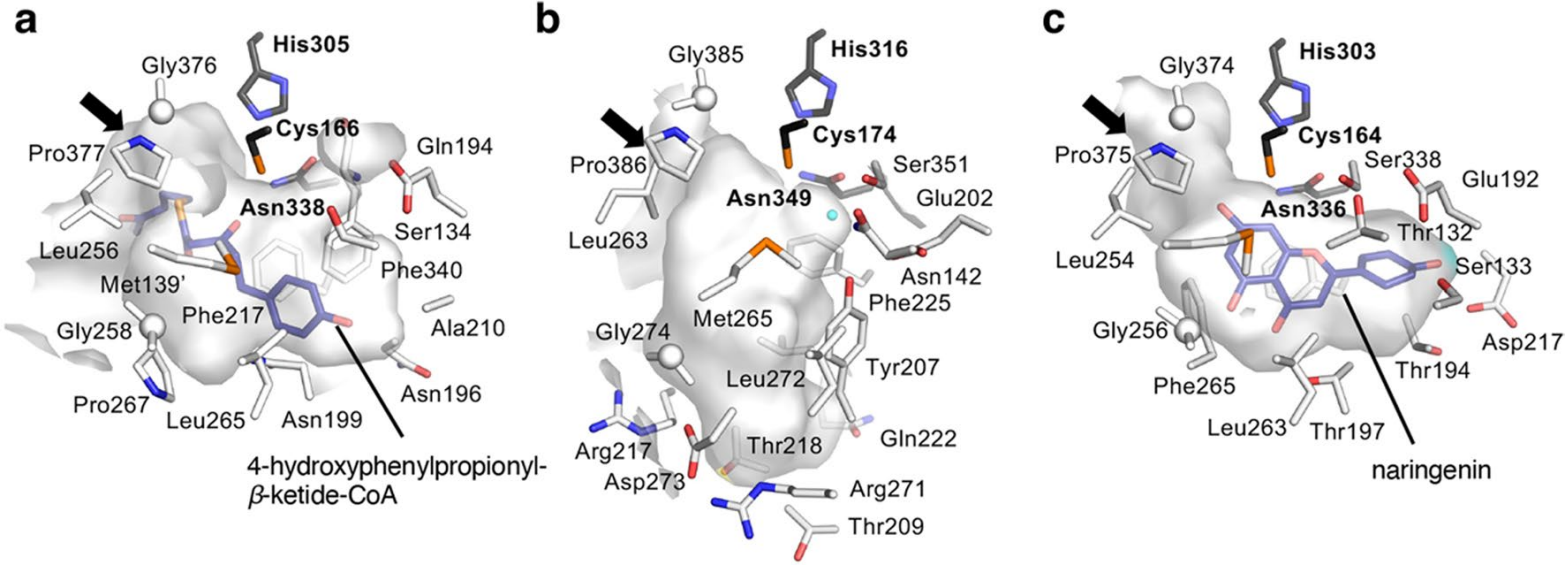
Comparison of the active-site architectures of **a** PECPS, **b** CUS, and **c** *Medicago sativa* CHS2. The 4-hydroxyphenylpropionyl-*β*-diketide-CoA from the docking simulation is shown as a blue stick model in the PECPS active-site cavity. The naringenin molecule complexed with the *M. sativa* CHS2 crystal structure and the water molecule in the Ser351-Asn142-H_2_O-Tyr207 rearrangement in CUS are indicated with a blue stick model and a light-blue sphere, respectively. The entrances of the active-site cavities are indicated with black arrows. The catalytic triads are highlighted with black stick models. Protein Data Bank ID numbers are as follows: PECPS, 7FFA; CUS, 3ALE; *M. sativa* CHS2, 1CGK

**Fig. 7:**
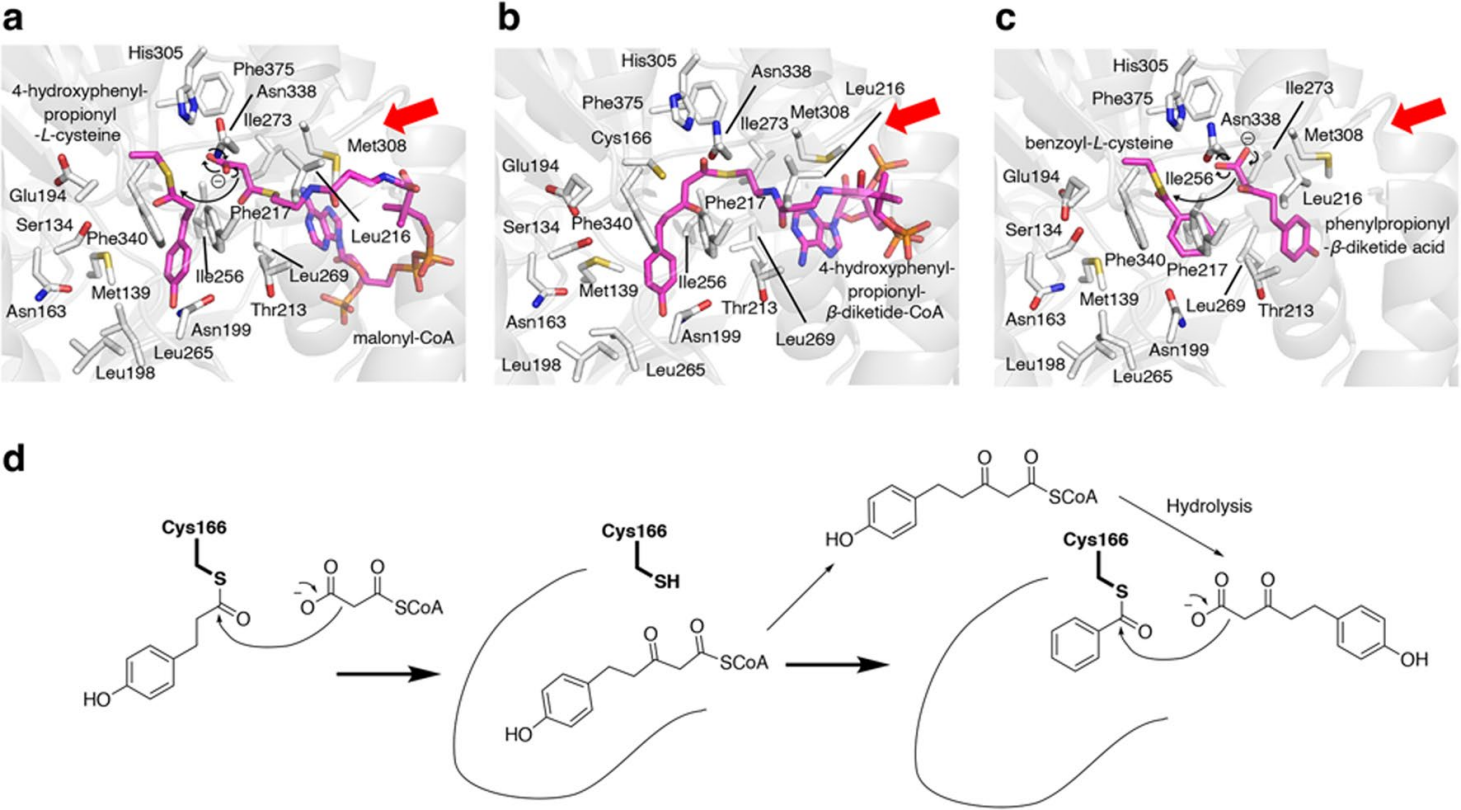
Proposed mechanism for the formation of 5-(4-hydroxyphenyl)-1-phenylpentane-1,3-dione by PECPS. Docking simulations of the **a** decarboxylative condensation of malonyl-CoA with the enzyme-bound 4-hydroxyphenylpropionyl unit, **b** formation of the 4-hydroxyphenylpropionyl-*β*-diketide-CoA intermediate, and **c** decarboxylative condensation of phenylpropionyl-*β*-diketide acid nonenzymatically derived from phenylpropionyl-*β*-diketide-CoA with the enzyme-bound benzoyl unit. **d** Schematic representation of the proposed mechanism for the formation of 5-(4-hydroxyphenyl)-1-phenylpentane-1,3-dione by PECPS. The entrances to the catalytic cavities are indicated with red arrows

Type III PKSs are homodimeric enzymes consisting only of keto synthase (KS) subunits, with molecular weights of about 40 kDa [38‒40, 43]. The crystal structure of PECPS showed the typical type III PKS fold, and no significant differences were observed in the overall structures [42]. However, the X-ray crystal structure revealed that PECPS lacks the pocket corresponding to the *β*-diketide acid intermediate-binding pocket in CUS [46], and instead, the active-site cavity of PECPS extends in a different direction from that of CUS (Fig. 6). The direction of the extended active-site cavity was similar to that of *Medicago sativa* CHS2 [51], but its depth was shallower. The observed active-site volume of the PECPS active-site cavity is thus apparently smaller than those of CUS and CHS, suggesting that it might be impossible for PECPS to accommodate the *β*-diketide acid intermediate with the second starter substrate in the active-site cavity as the second extender substrate. Furthermore, the docking studies suggested that the PECPS active-site cavity is large enough to accommodate the 4-hydroxyphenylpropionyl-*β*-diketide unit derived from the condensation of 4-hydroxyphenylpropionyl-CoA and malonyl-CoA (Figs. 6 and 7a‒c). However, it is not large enough to accept a benzoyl unit together with the 4-hydroxyphenylpropionyl-*β*-diketide unit, as expected (Fig. 7a‒c). In this docking simulation, the hydrogen bonding network containing the water molecule that might be responsible for the formation of a *β*-diketide acid intermediate like CUS was also absent in the PECPS active-site cavity. These results suggested that PECPS produces a diarylpentanoid by employing the following catalytic mechanism, which is completely different from that of CUS (Fig. 7d). (1) PECPS receives the 4-hydroxyphenylpropionyl unit of the initial starter substrate, 4-hydroxyphenylpropionyl-CoA, with the catalytic center Cys166, and then catalyzes the decarboxylative condensation of malonyl-CoA with the 4-hydroxyphenylpropionyl unit tethered to Cys166 to produce the 4-hydroxyphenylpropionyl-*β*-diketide-CoA intermediate. This is then released from the enzyme and hydrolyzed to 4-hydroxyphenylpropionyl-*β*-diketide acid. Subsequently, (2) PECPS receives the benzoyl unit of benzoyl-CoA with the catalytic center Cys166, and then condenses 4-hydroxyphenylpropionyl-*β*-diketide acid with the benzoyl unit, to generate 5-(4-hydroxyphenyl)-1-phenylpentane-1,3-dione as the final C_6_–C_5_–C_6_ product.

### Biosynthetic pathway of PECs

**Fig. 8:**
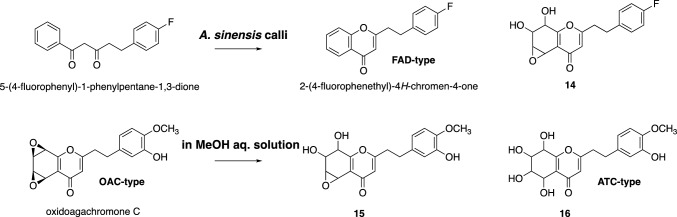
Structures of the compounds obtained from *A. sinensis* calli supplemented with 5-(4-fluorophenylethyl)-1-phenylpentane-1,3-dione, and the compounds derived from oxidoagalochromone C by maintaining it in a methanol solution

PECs are structurally classified into three types: FDC-type, OAC-type, and ATC-type, and the OAC-type has been proposed to be the biosynthetic precursor of the FDC- and ATC-types [52]. However, the present study clearly showed that the FDC-type is biosynthesized after the production of the diarylpentanoid by PECPS, although we did not identify any other enzymes involved in the biosynthesis after PECPS. Fortunately, biosynthetic pathways of compounds can often be predicted using substrate analogs. To this end, we also performed experiments feeding the non-physiological substrate, 5-(4-fluorophenylethyl)-1-phenylpentane-1,3-dione, to the *A. sinensis* calli. The feeding experiment revealed that the *A. sinensis* calli produced the FDC-type, 2-(4-fluorophenylethyl)-4*H*-chromen-4-one, together with its partially epoxidized compound **14** (Fig. 8). Furthermore, the stability test of oxidoagalochromone C in a methanol aqueous solution indicated that it was easily converted to compound **15** (structural analog of **14**) and compound **16** (all epoxy rings-cleaved OAC-type PEC) (Fig. 8). These findings suggested that among all three types, the FDC-type PECs are first biosynthesized from the linear diarylpentanoids via the hydroxylation and subsequent *O*-methylation(s) of the hydroxy group(s), and the OAC-type PECs are formed via the epoxidation of FDA-type PECs. Finally, the ATC-type PECs might be biosynthesized from OAC-type PECs by a ring‐opening reaction.

## Conclusion

The number of original agarwood plants has been drastically reduced due to excessive harvesting. Our research revealed the key enzyme in the biosynthesis of the agarwood fragrance components, PECs. Although the results are not immediately applicable to the cultivation of artificial agarwood, the discovery of PECPS as the key biosynthetic enzyme that forms the common intermediate of the PECs might facilitate explorations of different cultivation conditions by monitoring its gene expression. Although further identification of the downstream enzymes responsible for the biosynthesis of PECs after PECPS is also necessary, this foundational work on PECPS can be expanded to further create PECs analogs by incorporating the *pecps* gene into other species. This study thus provides insights into not only the cultivation of artificial agarwood, but also the conservation of original agarwood plants.

## Supplementary Information

Below is the link to the electronic supplementary material.

Supplementary file1 (PDF 185 KB)
