# Supplementary material for: Identification of a diarylpentanoid-producing polyketide synthase in the biosynthesis of 2-(2-phenylethyl)chromones in agarwood
Source: J Nat Med. 2023 Aug 19;77(4):667–76. doi: 10.1007/s11418-023-01743-5 (PMC10465673; doi:10.1007/s11418-023-01743-5)
Supplement: Supplementary file 1 — Supplementary file1 (PDF 185 KB) [file 11418_2023_1743_MOESM1_ESM.pdf]

Supplementary Material

Identification of a diarylpentanoid-producing polyketide synthase in the biosynthesis of 2-(2-phenylethyl)chromones in agarwood

Hiroyuki Morita[1*], Yuan-E Lee[1], She-Po Shi[2*]

[1] *Institute of Natural Medicine, University of Toyama, 2630-Sugitani, Toyama 930-0194, Japan.*

[2] *Modern Research Center for Traditional Chinese Medicine, Beijing University of Chinese Medicine, Beijing 100029, People's Republic of China.*

[*]Corresponding authors:

Hiroyuki Morita: Institute of Natural Medicine, University of Toyama, 2630-Sugitani, Toyama 930-0194, Japan. Tel.: +81-76-434-7625; email: hmorita@inm.u-toyama.ac.jp.

She-Po Shi: Modern Research Center for Traditional Chinese Medicine, Beijing University of Chinese Medicine, Beijing 100029, People's Republic of China; email: shishepo@163.com.

Contents

Figure S1: Possible PECPS reaction pathway to produce 5,7-dihydroxy-2-(4-hydroxyphenethyl)-4*H*-chromen-4-one from 4-hydroxyphenylpropionyl-CoA and malonyl-CoA.

Figure S2: Possible PECPS reaction pathway to produce 5-(4-hydroxyphenyl)-1-phenylpentane-1,3-dione from 4-hydroxyphenylpropionyl-CoA, benzoyl-CoA, and malonyl-CoA.

Figure S3: Possible PECPS reaction pathway to produce 5-(4-hydroxyphenyl)-1-phenylpentane-1,3-dione from 4-hydroxyphenylpropionyl-CoA, salicyloyl-CoA, and malonyl-CoA.

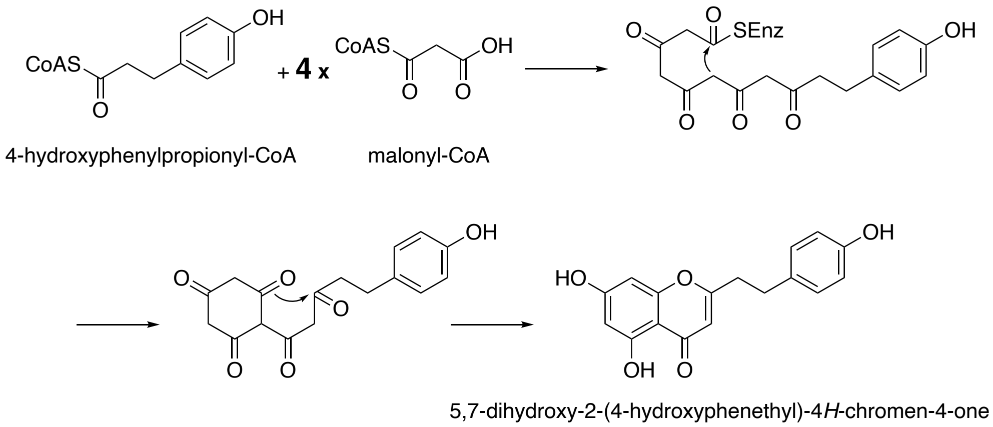

Figure S1: Possible PECPS reaction pathway to produce 5,7-dihydroxy-2-(4-hydroxyphenethyl)-4*H*-chromen-4-one from 4-hydroxyphenylpropionyl-CoA and malonyl-CoA.

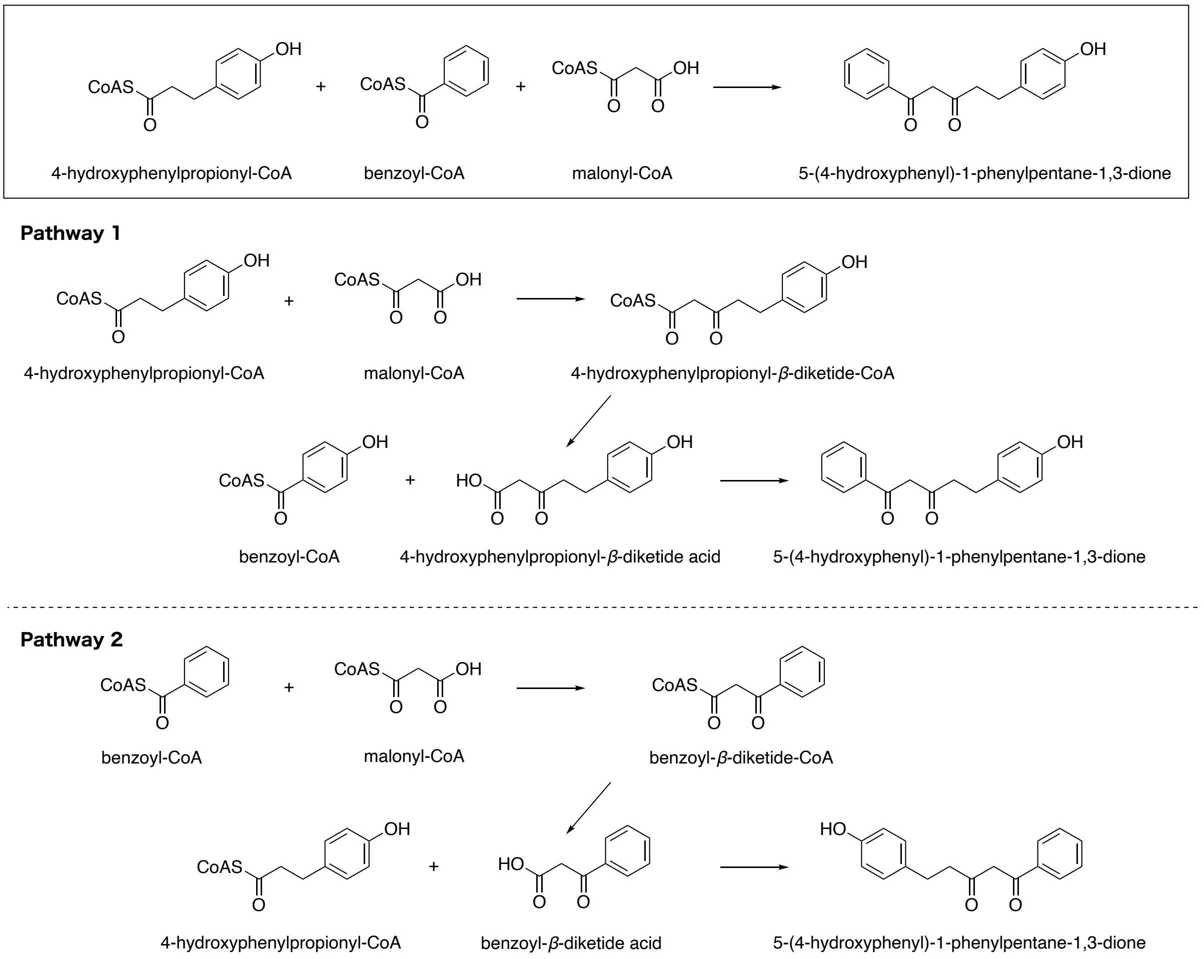

Figure S2: Possible PECPS reaction pathway to produce 5-(4-hydroxyphenyl)-1-phenylpentane-1,3-dione from 4-hydroxyphenylpropionyl-CoA, benzoyl-CoA, and malonyl-CoA.

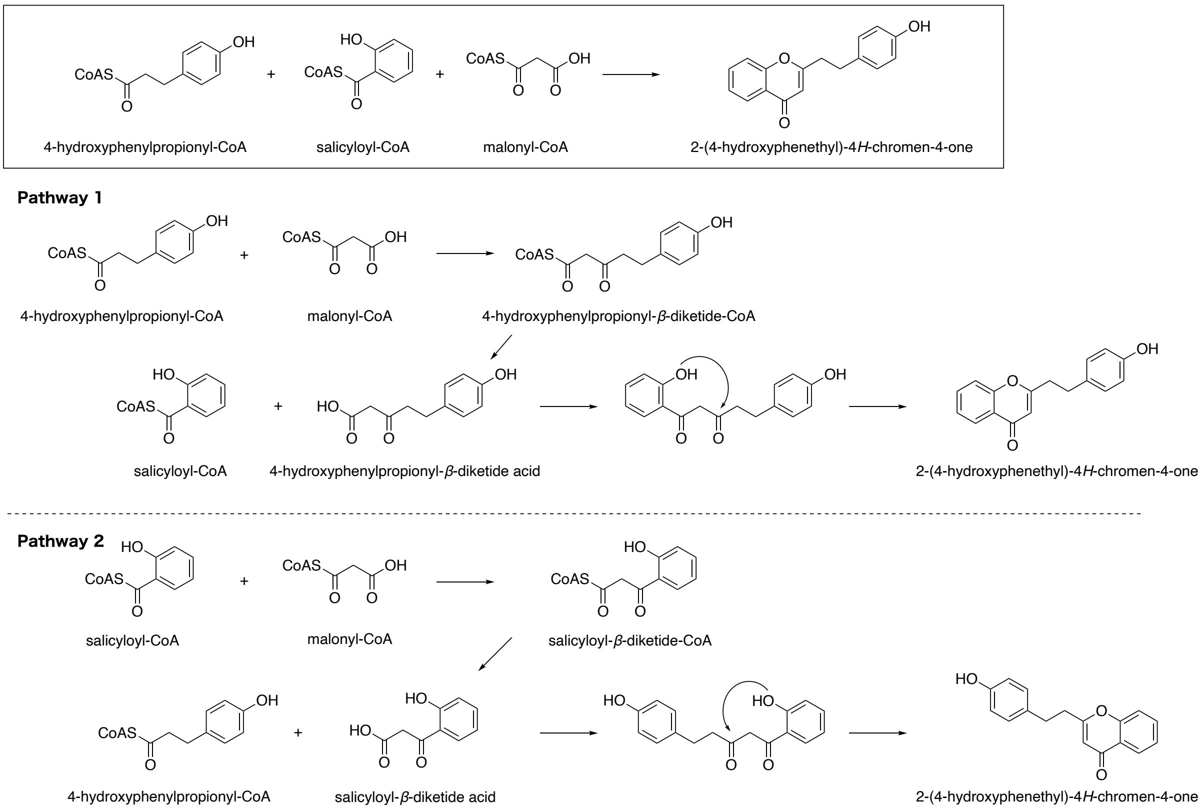

Figure S3: Possible PECPS reaction pathway to produce 5-(4-hydroxyphenyl)-1-phenylpentane-1,3-dione from 4-hydroxyphenylpropionyl-CoA, salicyloyl-CoA, and malonyl-CoA.
